# Mussel Adhesive Protein/Hyaluronic Acid Hydrogels for EGF Delivery and MRSA-Infected Diabetic Wound Repair

**DOI:** 10.3390/gels12060492

**Published:** 2026-06-02

**Authors:** Rong Tian, Han Yi, Jiaoyang Liu, Tong Wang, Tianyue Jiang, Song Qin

**Affiliations:** School of Pharmaceutical Sciences, Nanjing Tech University, Nanjing 211816, China; 13256437969@163.com (R.T.); 202361109009@njtech.edu.cn (H.Y.); liujiaoyang05@163.com (J.L.); 15189835663@163.com (T.W.); tjiang@njtech.edu.cn (T.J.)

**Keywords:** mussel adhesive protein, hydrogel dressing, diabetic wound healing, antibacterial, antioxidant

## Abstract

Diabetic foot ulceration is a severe and common chronic complication of diabetes, accompanied by excessive reactive oxygen species (ROS) accumulation, persistent bacterial infection, prolonged inflammation, and insufficient angiogenesis. Traditional single-function wound dressings fail to simultaneously resolve these pathological barriers, leading to unsatisfactory healing outcomes. In this study, we developed a multifunctional composite hydrogel (E/MGel) by introducing mussel adhesive protein (MAP) into methacrylated hyaluronic acid (mHA) to construct an antibacterial and antioxidant delivery system, which was further loaded with epidermal growth factor (EGF) to promote angiogenesis. The as-prepared E/MGel exhibited a uniform porous structure, favorable rheology, high swelling ratio, and sustained protein release behavior. In vitro results demonstrated that E/MGel exerted potent antibacterial activity against methicillin-resistant *Staphylococcus aureus* (MRSA) and *Escherichia coli* (*E.coli*), high ROS scavenging efficiency, good cytocompatibility, and remarkable pro-angiogenic effect on endothelial cells. In a mouse model of diabetic MRSA-infected full-thickness skin defect, E/MGel significantly accelerated wound closure, reduced bacterial burden, downregulated pro-inflammatory cytokines, promoted collagen deposition, and enhanced neovascularization. Meanwhile, no obvious systemic toxicity was observed. Taken together, this multifunctional hydrogel integrates antibacterial, antioxidant, and pro-angiogenic capacities to break the pathological vicious cycle of diabetic wounds, providing a promising and safe strategy for the clinical treatment of diabetic infected wounds.

## 1. Introduction

Diabetic foot ulcers are one of the most severe and common chronic complications of diabetes, leading to lower limb amputations and imposing a heavy healthcare burden [1,2]. The microenvironment of diabetic wounds is highly complex, and the core driver of impaired healing lies in the redox imbalance induced by persistent hyperglycemia [3]. Specifically, the excessive accumulation of reactive oxygen species (ROS), including superoxide anions (O_2_^−^) and hydroxyl radicals (·OH), not only directly attacks lipids, proteins, and DNA, causing cellular dysfunction and apoptosis, but also significantly prolongs the inflammatory cycle, inhibits growth factor activity, and facilitates the colonization of pathogenic bacteria and biofilm formation [4,5,6]. Notably, bacterial colonization and biofilm formation further exacerbate local inflammatory responses, leading to the overexpression of pro-inflammatory cytokines such as Tumor Necrosis Factor-alpha (TNF-α) and Interleukin-6 (IL-6) [7,8]. In turn, excessive inflammatory mediators stimulate the continuous production of ROS, creating a vicious cycle [9]. Under these pathological conditions, fibroblast proliferation and collagen synthesis are suppressed, and angiogenesis is impeded. The wound remains stagnant in the persistent inflammatory phase, unable to transition smoothly to the proliferation and remodeling phases, ultimately resulting in chronic, non-healing ulcers. Consequently, the synergistic elimination of excessive ROS, inhibition of bacterial colonization, and disruption of the inflammatory vicious cycle have become key strategies for promoting wound healing in diabetes [10].

Traditional single-function dressings (such as gauze, foam, and conventional gels) can only provide passive protection and struggle to simultaneously address infection, oxidative stress, and impaired angiogenesis, often leading to prolonged non-healing of wounds [11,12,13]. In recent years, hydrogels have emerged as ideal multifunctional dressing carriers due to their biomimetic three-dimensional network, excellent biocompatibility, tunable mechanical properties, and high loading capacity [14,15]. Hydrogels not only maintain wound moisture and absorb exudate but also serve as multifunctional carriers capable of flexibly loading exogenous or endogenous active substances. By integrating components such as antioxidants, antibacterial agents, and angiogenesis promoters, they enable active regulation of the wound microenvironment [16,17,18]. Currently, the functional components under intensive research primarily fall into two categories: exogenous active substances (such as plant polyphenols, antibiotics, and metal nanoparticles [19,20,21]), which are widely available; among these, antioxidants (such as curcumin and resveratrol) can promote healing through mechanisms such as scavenging reactive oxygen species and activating the Nrf2 pathway [22,23,24]; antimicrobial strategies include loading antibiotics, incorporating inorganic nanoparticles (such as silver nanoparticles), and utilizing natural antimicrobial substances (chitosan, polylysine, etc.) [25,26,27]. Endogenous active substances (such as superoxide dismutase, glutathione, melatonin, the antimicrobial peptide LL-37, and lactoferrin) have attracted significant attention due to their high biocompatibility and low immunogenicity. Their hydrogel delivery systems can compensate for insufficient endogenous levels under pathological conditions and show great promise in scavenging free radicals, inhibiting bacteria, and promoting angiogenesis [24,28,29,30,31,32].

Among the many bioactive materials, mussel adhesive protein (MAP) derived from marine mussels has attracted significant attention due to its unique properties [33]. MAP is rich in 3,4-dihydroxyphenylalanine (Dopa) and lysine (Lys) residues, which confer multiple functions: the catechol group of Dopa exerts antioxidant effects by scavenging free radicals and chelating transition metal ions [34,35,36,37,38]; Lys residues carry a positive charge under physiological conditions and can disrupt bacterial cell membranes through electrostatic adsorption, thereby achieving broad-spectrum antibacterial activity against MRSA. Furthermore, as a natural protein, MAP exhibits excellent biocompatibility and degradability [39], making it an ideal multifunctional molecule for constructing complex wound treatment platforms.

Growth factors (GFs) play a critical role in wound healing by stimulating cell migration, proliferation, and differentiation [40]. The U.S. Food and Drug Administration (FDA) has approved multiple growth factor-containing products for clinical use, confirming the feasibility of this approach [41]. Among these, epidermal growth factor (EGF) is a key factor in promoting angiogenesis; by binding to its receptor, it specifically promotes endothelial cell tube formation, which helps address the issue of insufficient angiogenesis in diabetic wounds [42]. However, EGF has a short half-life in vivo and is easily degraded by proteases. Traditional formulations suffer from shortcomings such as short local retention time and susceptibility to being washed away by wound exudate. When used alone, its bioavailability is extremely low, and its biological effects are unstable due to concentration fluctuations [43]. Therefore, there is an urgent need for a carrier strategy that can protect EGF activity and enable its stable local delivery.

**Scheme 1:**
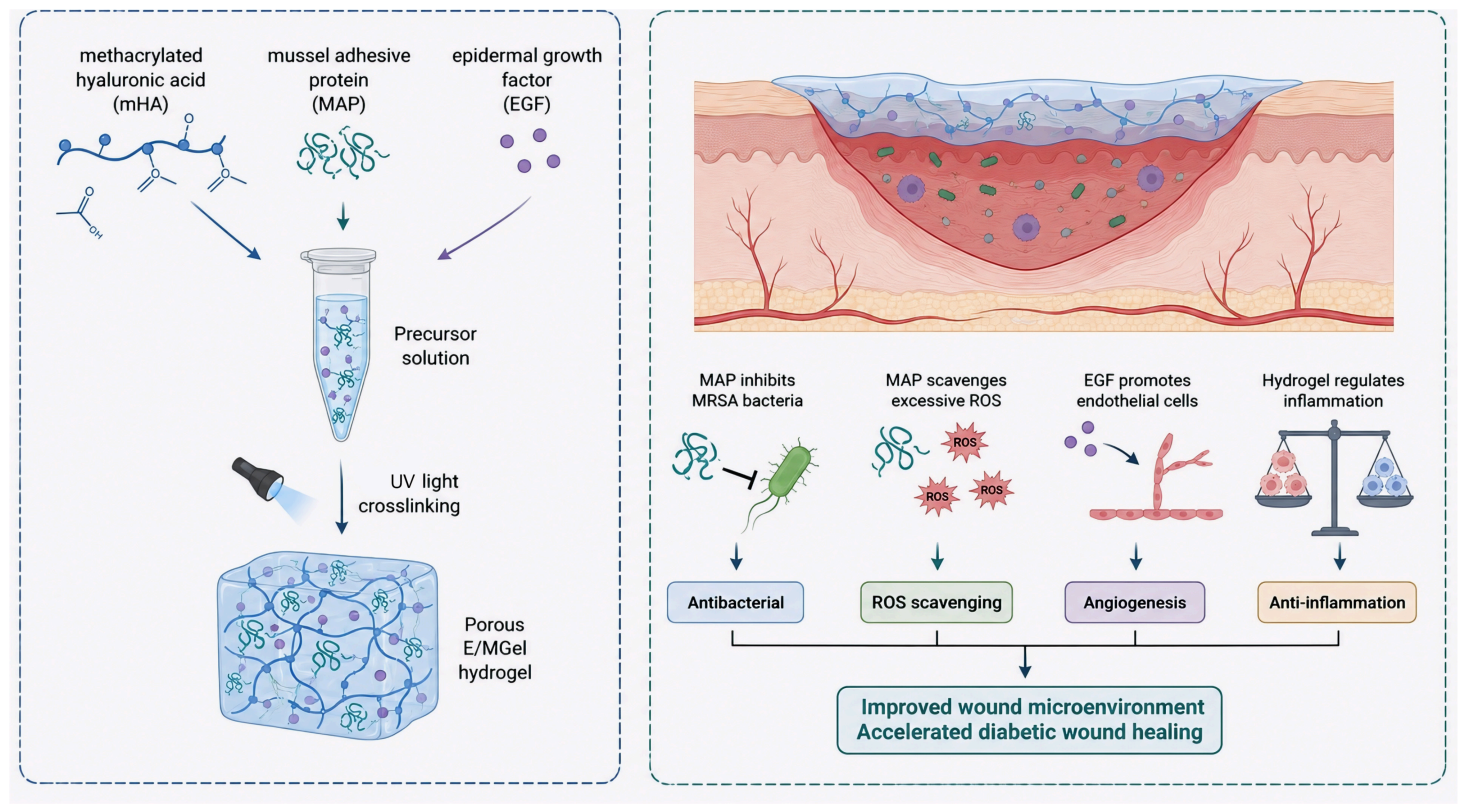
Schematic illustration of the E/MGel hydrogel system for diabetic wound healing. The hydrogel is prepared from mHA, MAP, and EGF via UV crosslinking, and exerts antibacterial, antioxidant, pro-angiogenic, and anti-inflammatory effects to accelerate diabetic wound healing.

Based on the above analysis, this study aims to develop a multifunctional hydrogel dressing that integrates antibacterial, antioxidant, and angiogenic properties to promote the healing of diabetic infected wounds (Scheme 1). Specifically, we first incorporated mussel adhesive protein (MAP), which possesses both broad-spectrum antimicrobial and potent antioxidant activities, into a methylacrylated hyaluronic acid hydrogel matrix to construct a multifunctional delivery carrier with antimicrobial and antioxidant properties; we then loaded EGF, a key factor promoting angiogenesis, onto this carrier, ultimately preparing a composite hydrogel dressing (E/MGel). We systematically investigated the physicochemical properties (microstructure, swelling behavior, rheological properties, and oxidative-responsive release characteristics), biocompatibility (cytotoxicity, hemolysis), and in vitro functional properties (scavenging capacity for hydroxyl radicals and superoxide anions, antibacterial activity against MRSA and *E. coli*, and promotion of tube formation in human umbilical vein endothelial cells) and further evaluated its comprehensive in vivo efficacy—including wound closure rate, bacterial clearance efficiency, inflammation regulation, collagen deposition, and vascular regeneration—by establishing a mouse model of infected full-thickness skin defects on the back. This study aims to provide a novel, highly effective, and safe material solution for the clinical treatment of hard-to-heal infected wounds, such as those associated with diabetes, through this multi-component synergistic strategy.

## 2. Results and Discussion

### 2.1. Preparation and Characterization of E/MGel Hydrogel

**Figure 1:**
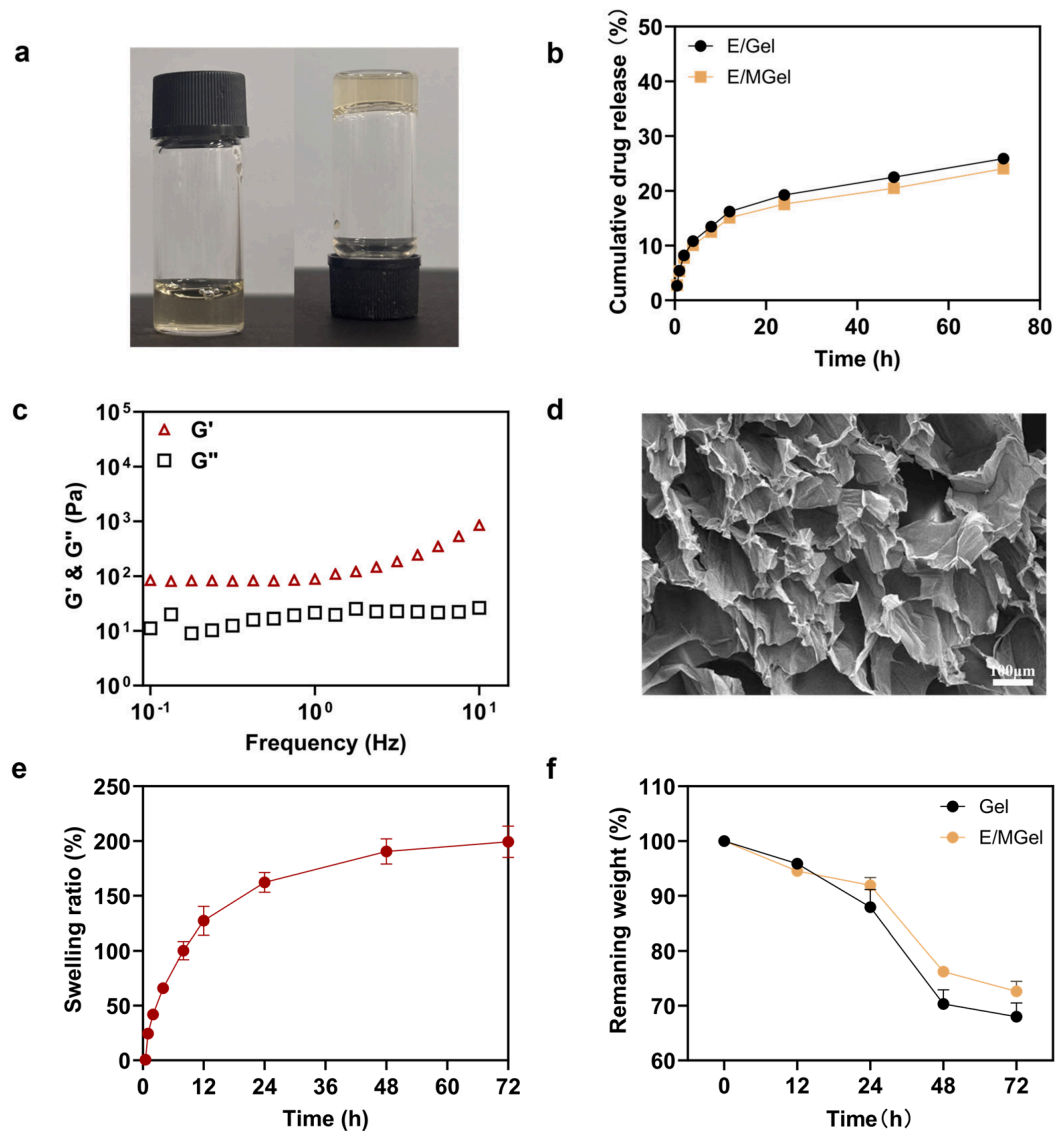
(**a**) Image of E/MGel hydrogel. (**b**) Drug release behavior of E/Gel and E/MGel hydrogel. (**c**) Rheological performance test under the dynamic frequency scanning mode of E/MGel hydrogel. (**d**) SEM image of E/MGel hydrogel. (**e**) Swelling performance of E/MGel hydrogel in PBS. (**f**) In vitro degradation profiles of Gel and E/MGel hydrogel.

The structure of the product was confirmed by ^1^H NMR. The characteristic peaks at 5.75 ppm and 6.19 ppm in the spectrum belonged to the double bond hydrogen in the methacrylic acid structure. By integrating the peak areas, the grafting rate of the HA double bond was approximately 12.5% (Figure S1). The macroscopic morphology of E/MGel hydrogel is shown in (Figure 1a). Both E/Gel and E/MGel exhibited sustained EGF release over 72 h without excessive burst (Figure 1b). At 2 h, release was similar (~7.8%). From 12 h onward, E/MGel showed lower cumulative release than E/Gel (24 h: 17.59 ± 0.76% vs. 19.27 ± 0.11%; 72 h: 24.04 ± 0.80% vs. 25.88 ± 0.12). The slow release observed in E/MGel may be attributed to the electrostatic interaction between membrane proteins (MAP) and EGF, which supports a stable release pattern suitable for chronic wound healing. Rheological analysis demonstrated that the storage modulus (G′) was consistently higher than the loss modulus (G″) over the tested frequency range, verifying the formation of a stable elastic network (Figure 1c). SEM images revealed that the lyophilized hydrogel possessed a continuous, interconnected 3D porous network with a pore size of 50–150 μm, which favors cell infiltration and nutrient transport (Figure 1d). Figure 1e shows an outstanding swelling ratio of ~200% within 72 h in PBS, enabling efficient exudate absorption and maintenance of a moist wound microenvironment. The degradation of Gel and E/MGel hydrogels was evaluated by measuring remaining dry weight after incubation in PBS containing 100 U/mL hyaluronidase at 37 °C for up to 72 h (Figure 1f). Both hydrogels exhibited time-dependent degradation: Gel retained 87.9 ± 2.8% and 68.0 ± 2.1% of its initial mass at 24 h and 72 h, respectively, while E/MGel retained significantly higher mass (91.9 ± 1.2% at 24 h; 72.8 ± 1.4% at 72 h), indicating that MAP incorporation retarded enzymatic degradation. Nevertheless, this study only characterized short-term in vitro enzymatic degradation within 72 h. The long-term degradation profile under physiological conditions, in vivo degradation kinetics, and associated long-term biosafety remain to be systematically explored to fully support clinical translation.

### 2.2. Antibacterial and Antioxidant Performance

**Figure 2:**
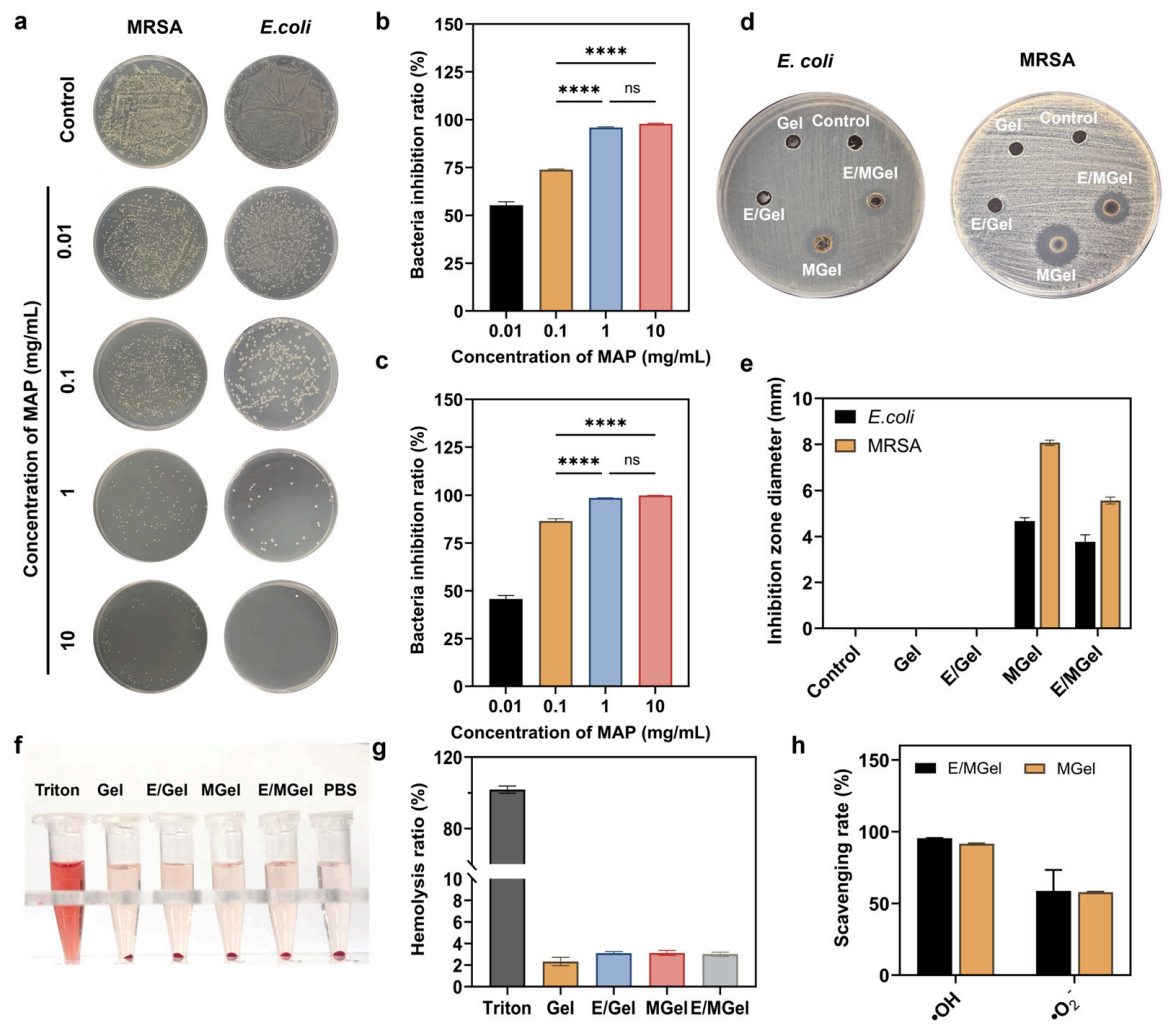
(**a**) Representative images of MRSA and *E. coli* colonies after treatment with different concentrations (0.01, 0.1, 1, 10 mg/mL) of MAP. (**b**) Data on bacterial inhibition rates of MAP against MRSA and (**c**) *E. coli*, expressed as mean ± standard deviation (**** *p* < 0.0001, ns: no significant difference). (**d**) Representative images of the Oxford cup antibacterial test for *E. coli* and MRSA. (**e**) Corresponding inhibition zone diameter data, expressed as mean ± standard deviation. (**f**) Representative photos of hemolysis test. (**g**) Quantitative hemolysis rates of different hydrogel extracts. (**h**) ·OH and O_2_^−^ free radical scavenging rates of E/MGel and MGel.

Figure 2a shows the colony morphology of *E. coli* and MRSA after treatment with MAP at different concentrations. Colony counts decreased markedly in a concentration-dependent manner. Low-dose MAP (0.01, 0.1 mg/mL) significantly reduced colony formation, while almost no colonies survived at 10 mg/mL. Quantitative antibacterial rates (Figure 2b,c) were 45.68%, 86.54%, 98.57%, 99.91% for *E. coli* and 55.23%, 73.87%, 95.90%, 97.84% for MRSA at 0.01–10 mg/mL, with significant differences between low- and medium/high-concentration groups (*p* < 0.0001). These results confirm the potent, concentration-dependent broad-spectrum antibacterial activity of MAP against *E. coli* and MRSA. The antibacterial properties of Gel, E/Gel, MGel, and E/MGel were further evaluated by inhibition zone assay (Figure 2d,e). No inhibition zones were observed in Control, Gel, or E/Gel groups. In contrast, MGel showed strong antibacterial activity, with inhibition zone diameters of 4.67 ± 0.15 mm against *E. coli* and 8.07 ± 0.12 mm against MRSA. E/MGel also exhibited significant activity (3.77 ± 0.31 and 5.57 ± 0.15 mm, respectively). The slightly lower activity of E/MGel may result from electrostatic interactions between positively charged Lys residues of MAP and negatively charged EGF. These results confirm MAP as the core antibacterial component. Notably, these in vitro antibacterial results may not fully represent efficacy against established biofilm infections in the complex diabetic wound microenvironment. MAP is rich in dopamine and lysine residues, conferring both broad-spectrum antibacterial and antioxidant activities by disrupting bacterial membranes and scavenging free radicals [44]. Mechanistic studies further revealed that MGel induced time-dependent increases in conductivity, reaching approximately 21 μS/cm for MRSA and 20 μS/cm for *E. coli* at 12 h, as well as a marked increase in A_260_ (0.3–0.4) compared with the PBS and Gel groups (Figures S2 and S3). These results demonstrate that MAP exerts antibacterial effects by disrupting bacterial membrane integrity, leading to ion and nucleic acid leakage.

As shown in Figure 2f,g, all hydrogel formulations showed hemolysis rates below the 5% safety criterion, confirming favorable hemocompatibility and no obvious hemolytic toxicity.

To assess the in vitro antioxidant capacity of hydrogels, the scavenging efficiencies of typical reactive oxygen species (ROS), including hydroxyl radical (·OH) and superoxide anion (·O_2_^−^), were determined in each group. As presented in Figure 2h, the clearance rates of MGel for ·OH and ·O_2_^−^ were 91.58% and 57.75%, respectively. Furthermore, E/MGel fabricated by further loading EGF showed slightly elevated scavenging rates (95.48% for ·OH and 58.67% for ·O_2_^−^), with no statistical difference compared with MGel. These results suggested that MAP was the key component endowing hydrogels with high-efficiency antioxidant activity and could markedly strengthen their scavenging capacity against various ROS. Meanwhile, the incorporation of EGF did not interfere with or weaken the ROS-scavenging effect of hydrogels, providing an important basis for subsequent application in diabetic wounds under high oxidative stress.

### 2.3. Cytocompatibility and Pro-Angiogenic Ability

**Figure 3:**
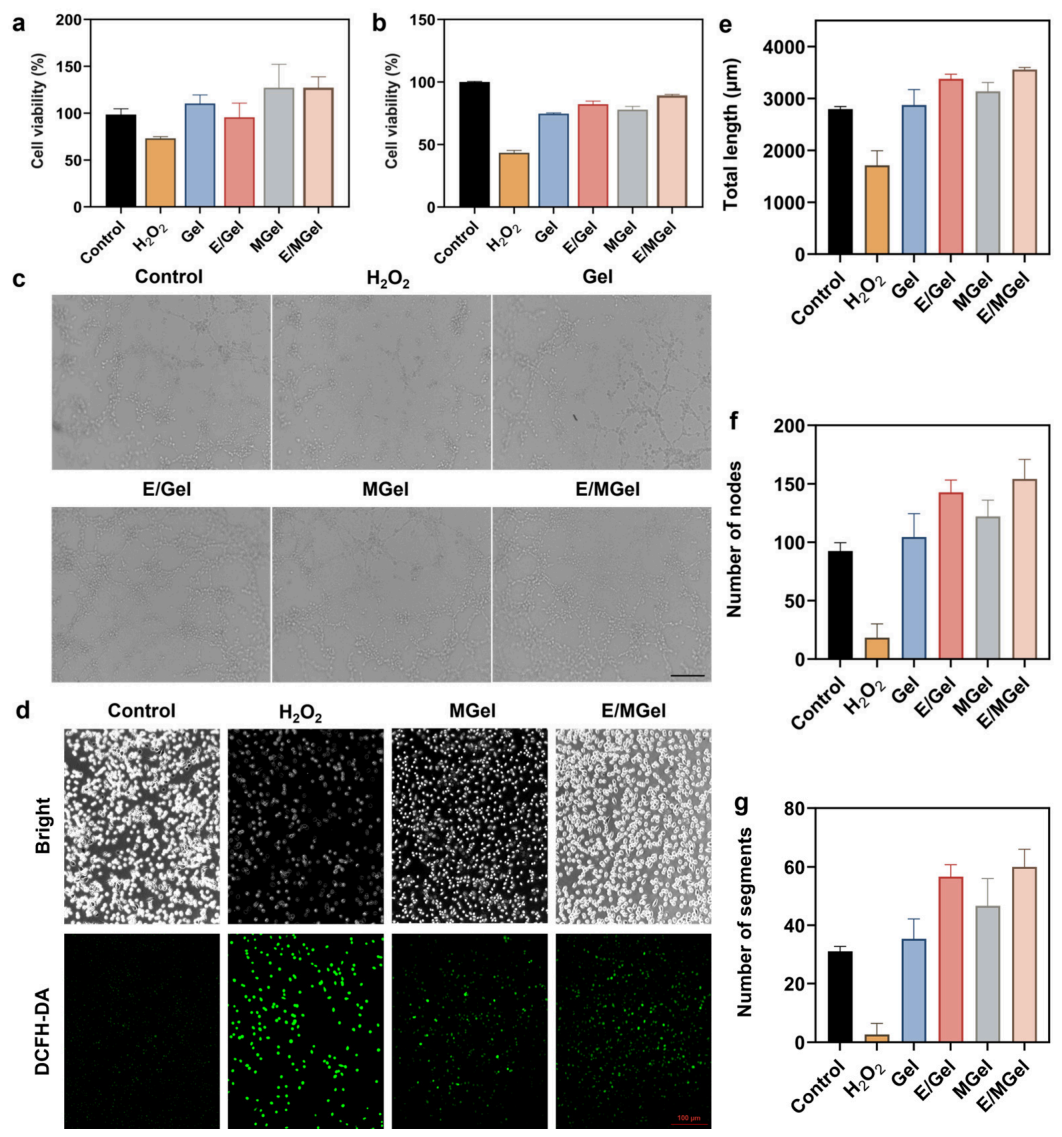
Cell viability of different hydrogels after incubation with (**a**) L929 cells and (**b**) HUVEC cells for 24 h. (**c**) A typical schematic diagram of the tubular structure formed by HUVEC cells after 4 h of in vitro culture, with a scale of 200 μm. (**d**) Bright-field images (top) and DCFH-DA fluorescence staining images (bottom) of L929 cells after incubation with different gel groups in an environment of 0.3 mM H_2_O_2_, with a scale of 100 μm. Quantitative statistics of the tube formation experiment with HUVEC cells: (**e**) Total tube length, (**f**) Number of nodes, (**g**) Number of segments.

Excellent biocompatibility is essential for hydrogel wound dressings. In this study, CCK-8 assays were used to evaluate the effects of Gel, MGel, E/Gel, and E/MGel extracts on the viability of L929 fibroblasts and HUVECs under 0.3 mM H_2_O_2_-induced oxidative stress for 24 h. As shown in Figure 3a,b, compared with the blank control, cell viability was significantly reduced to below 80% in the H_2_O_2_ model group, confirming successful oxidative stress injury. In contrast, all hydrogel-treated groups exhibited remarkably restored cell viability above 80%. In particular, the E/MGel group showed the highest viability, which was significantly higher than the H_2_O_2_ model group. Notably, cell viability in some hydrogel groups slightly exceeded 100%, indicating a mild pro-proliferative effect rather than cytotoxicity. These results demonstrate that the hydrogels have favorable biocompatibility and can effectively protect cells from oxidative damage, providing a safe microenvironment for wound healing. Angiogenesis is a key step in wound healing; accelerated vascular formation effectively promotes wound repair. In this study, an in vitro tube formation assay using human umbilical vein endothelial cells (HUVECs) was performed to evaluate the pro-angiogenic ability of different hydrogels. As shown in Figure 3c, compared with the normal control group, the H_2_O_2_-treated group displayed sparse, broken and incomplete tubular structures, indicating significantly inhibited tube formation. The Gel group formed a relatively continuous vascular network, with a notable improvement compared with the H_2_O_2_ group. The E/Gel and MGel groups exhibited further enhanced vascular networks with increased branches. Notably, the E/MGel group showed the optimal tube formation effect, with intact vascular networks, clear lumens and dense branches, which were significantly superior to those of the pristine hydrogel and single-component modified groups.

Quantitative analysis (Figure 3e–g) demonstrated that total tube length, number of nodes and segments were significantly decreased in the H_2_O_2_-treated group compared with the control group, confirming that oxidative stress markedly inhibits endothelial tube formation. All tube formation parameters in the Gel group were significantly elevated relative to the H_2_O_2_ group, suggesting that the bare hydrogel matrix partially restored cellular angiogenic activity. Furthermore, E/Gel and MGel groups exhibited further improvements in these indices. Among all groups, E/MGel achieved the highest values of total tube length, node number and segment number, which were superior to those of Gel and single-modified hydrogels, fully verifying its optimal pro-angiogenic capacity.

Oxidative stress is one of the core pathological characteristics responsible for the refractory healing of diabetic chronic wounds. Excessive accumulation of reactive oxygen species (ROS) aggravates cell injury, prolongs inflammatory responses, and severely impairs wound repair. The intracellular antioxidant capacity of hydrogels was evaluated by DCFH-DA staining. As displayed in Figure 3d, the normal control group showed only extremely low green fluorescence, indicating a low basal ROS level. The model group stimulated with 0.3 mM H_2_O_2_ exhibited intense and widespread green fluorescence, confirming the successful establishment of the oxidative stress injury model. Compared with the model group, the intracellular fluorescence intensity was significantly reduced after treatment with MGel and E/MGel hydrogels.

### 2.4. In Vivo Diabetic Infected Wound Healing

Previous in vitro studies have verified that E/MGel hydrogel possesses excellent physicochemical properties, biocompatibility and biological activity. To further assess its in vivo therapeutic effect, a diabetic mouse model was induced by streptozotocin (STZ), and a full-thickness MRSA-infected skin defect was established on the back for in vivo evaluation.

**Figure 4:**
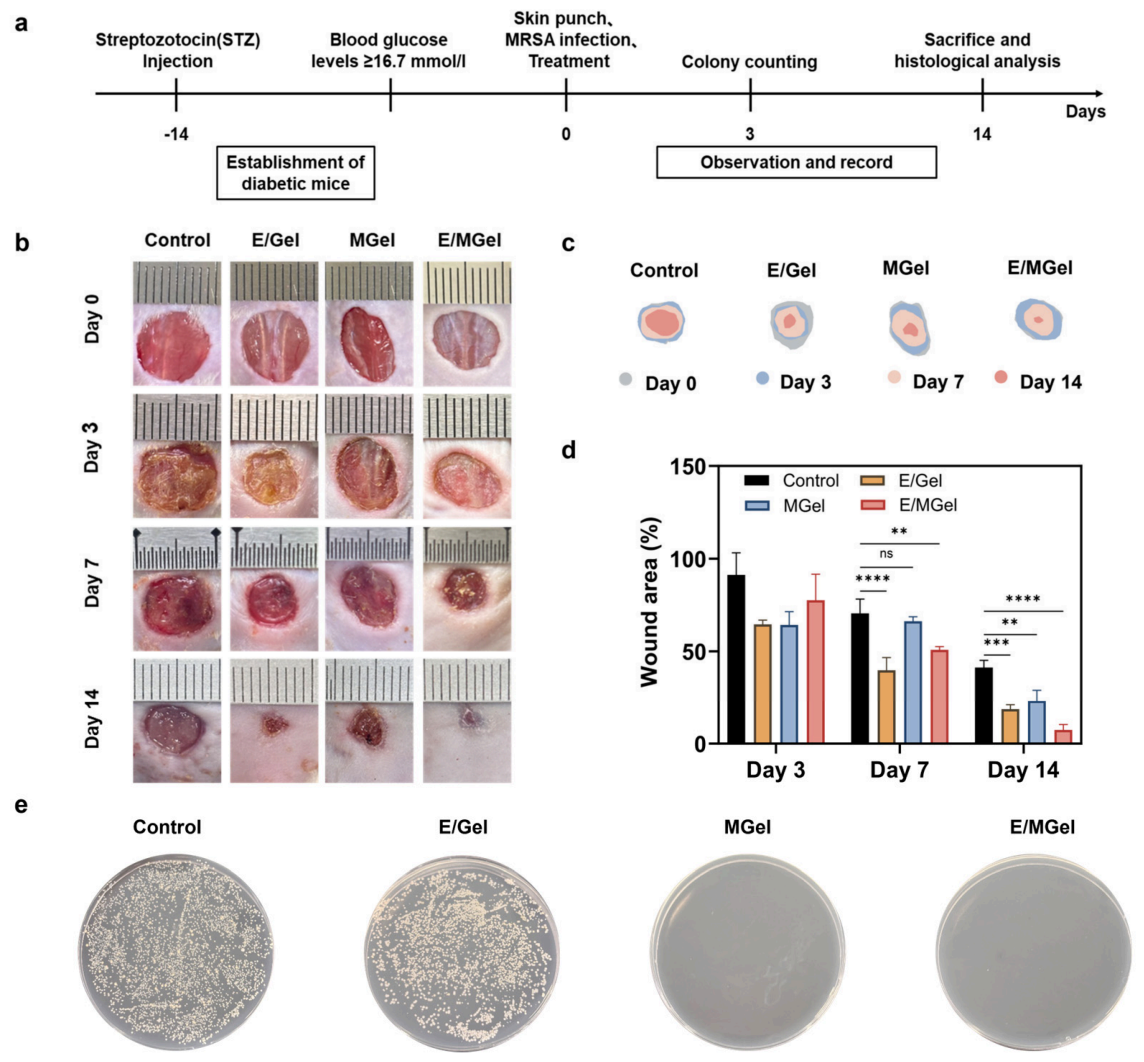
(**a**) Flowchart of in vivo experiment. (**b**) Wound healing morphology of each group of mice on the 0th, 3rd, 7th, and 14th days after modeling. (**c**) Schematic diagram of the wound contour at corresponding time points, with different colors marking the wound boundaries at each time point. (**d**) Quantitative statistical results of the percentage of remaining wound area in each group, ** *p* < 0.01, *** *p* < 0.001, **** *p* < 0.0001. ns = not significant. (**e**) Representative images of MRSA colonies collected from the wounds of diabetic mice on the 3rd day after different hydrogel treatments.

As shown in Figure 4a, the experimental procedure included diabetic model establishment, MRSA-infected wound construction, hydrogel treatment, and analyses on days 3 and 14. General observation (Figure 4b,c) and quantitative analysis (Figure 4d) revealed that the E/MGel group exhibited significantly accelerated wound closure as early as day 3, with only 8.72% of the wound area remaining on day 14, markedly superior to the control, E/Gel, and MGel groups. While the mouse model cannot fully recapitulate the impaired peripheral circulation characteristic of human diabetic patients, these results indicate that the early therapeutic benefit was mainly attributed to the antibacterial effect of MAP, which inhibited MRSA and alleviated excessive inflammation. Figure 4e shows the representative MRSA colony images isolated from diabetic wound tissues in different groups. Abundant bacterial colonies were observed in the Control and E/Gel groups, demonstrating severe persistent bacterial infection. In contrast, extremely few colonies existed in the MGel group, and nearly no visible MRSA colonies were detected in the E/MGel group. These results verify the excellent in vivo antibacterial efficacy of MAP-functionalized hydrogels against MRSA-infected diabetic wounds.

To systematically evaluate the effect of the E/MGel hydrogel on healing quality in diabetic mice, wound tissues were harvested on day 14 post-surgery and subjected to H&E staining and Masson’s trichrome staining. Analysis was performed from the perspectives of histological structure and matrix remodeling. In H&E staining, pink regions represented cytoplasm and extracellular matrix, and blue-purple regions indicated hematoxylin-counterstained nuclei. The epidermal, dermal, and granulation tissue layers could be clearly distinguished. More intact histological structure, clearer stratification, and more mature granulation tissue indicated better wound healing quality.

**Figure 5:**
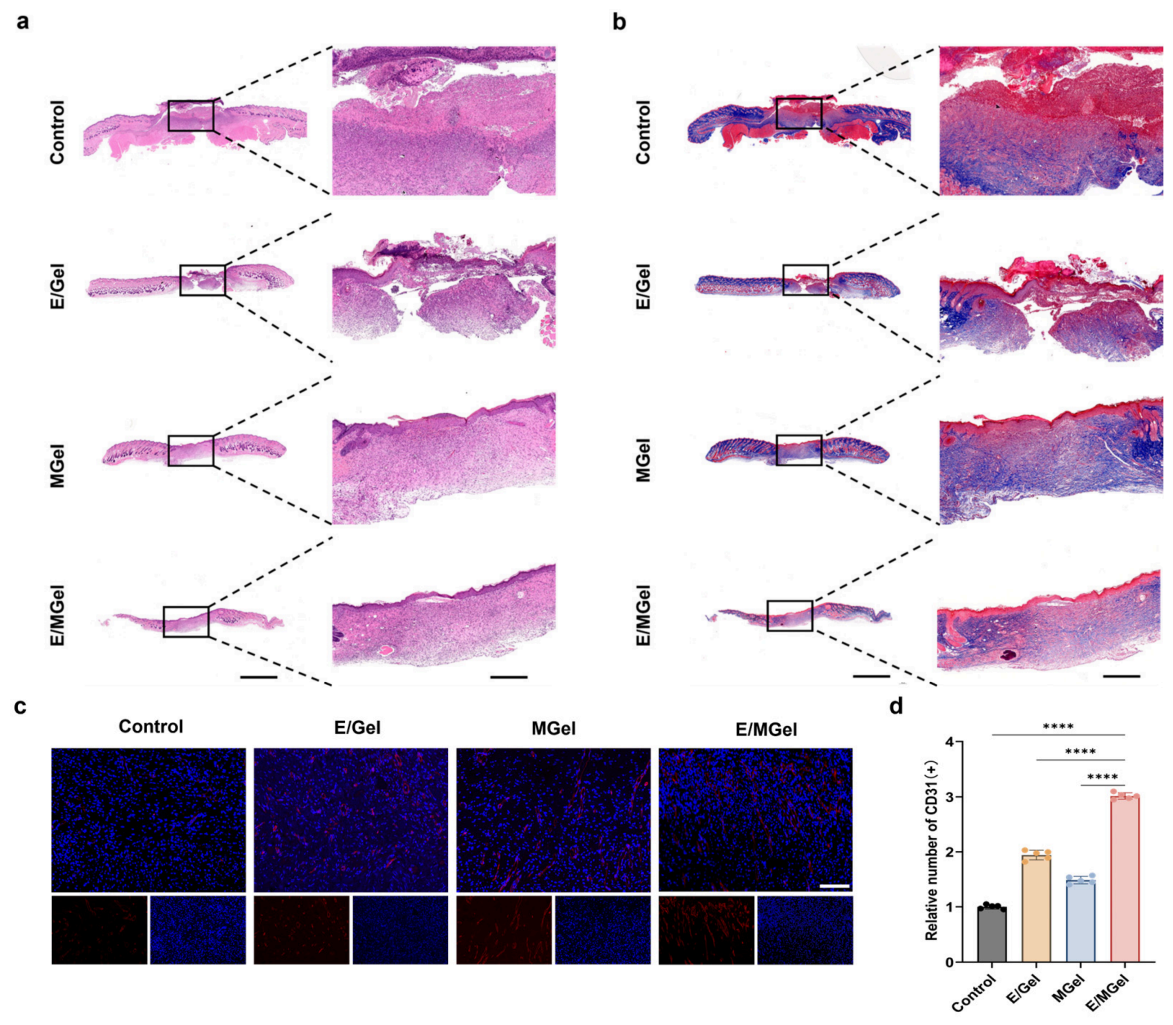
(**a**) H&E staining image of the wound tissue of diabetic mice after 14 days of hydrogel treatment (scale = 500 μm), and (**b**) Masson staining image (scale = 500 μm). (**c**) Representative image of CD31 immunofluorescence staining of wound tissue in diabetic mice 14 days later (red: CD31, blue: DAPI, scale: 100 μm). (**d**) The relative number of CD 31^+^ cells. Compared with the E/MGel group, **** *p* < 0.0001, *n* = 5.

As shown in Figure 5a, the control group exhibited incomplete epidermal and dermal repair, insufficient tissue regeneration, mild inflammatory infiltration and immature granulation tissue, while the E/Gel and MGel groups showed improved epidermal regeneration but still presented disordered dermal structure and underdeveloped granulation tissue. In contrast, the E/MGel group displayed well-differentiated epidermal and dermal layers with mature granulation tissue. According to Masson’s trichrome staining (Figure 5b), the control group had low, loose and immature collagen deposition, the E/Gel and MGel groups showed increased but irregularly distributed collagen with limited maturity, and the E/MGel group demonstrated significantly higher collagen content with dense, regular and mature collagen bundles, indicating superior wound healing quality.

To assess angiogenesis during wound healing, CD31 immunofluorescence staining was used to label neovascular endothelial cells. CD31 (platelet endothelial cell adhesion molecule-1) is a specific marker for vascular endothelial cells, and its expression reflects vascular density and neovascularization. As shown in Figure 5c, red fluorescence indicated CD31-positive expression (endothelial cells), and blue fluorescence represented DAPI-stained nuclei. On day 14 post-surgery, varying degrees of CD31-positive signals were observed in all groups. The control group showed weak and scattered red fluorescence with sparse positive areas, indicating limited neovascularization and an incomplete vascular network. The E/Gel and MGel groups exhibited partial continuous tubular structures but relatively low fluorescence intensity. In contrast, the E/MGel group showed the brightest red fluorescence, greatly expanded positive areas, and abundant reticular connections, indicating a denser neovascular network.

**Figure 6:**
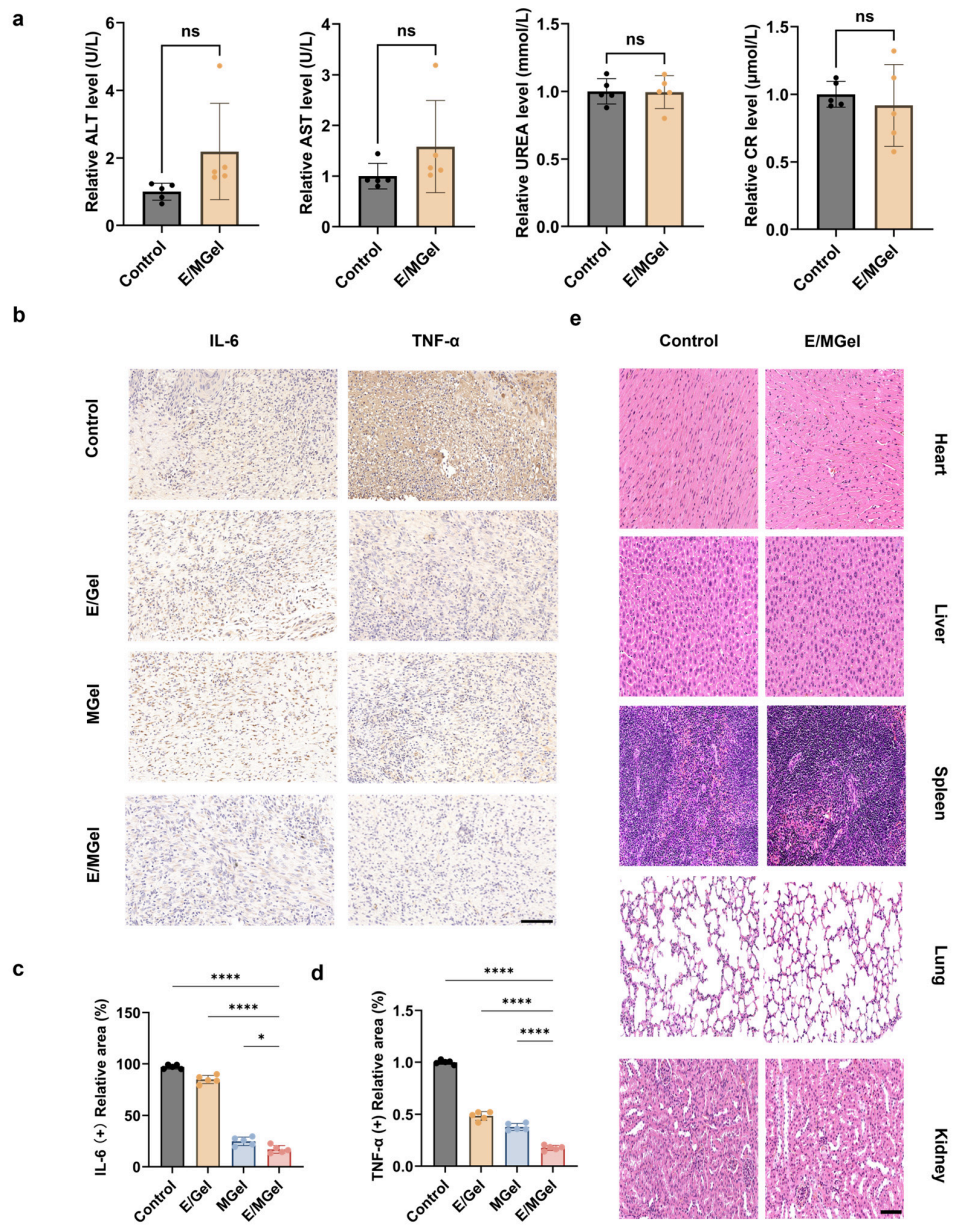
(**a**) Serum biochemical markers of liver (ALT, AST) and kidney (CR, UREA) function after 14 days of treatment (*n* = 5, ns: not significant). (**b**) Immunohistochemical staining of IL-6 and TNF-α in wound tissues on day 14 (scale bar = 100 μm). (**c**,**d**) Quantitative analysis of IL-6 (**c**) and TNF-α (**d**) positive areas in wound tissues (* *p* < 0.05, **** *p* < 0.0001). (**e**) H&E staining of heart, liver, spleen, lung and kidney tissues (scale bar = 500 μm).

Persistent excessive inflammation often leads to delayed diabetic wound healing. To investigate the hydrogel’s regulatory effect on the inflammatory microenvironment, IL-6 and TNF-α were detected by immunohistochemistry on day 14. Brown positive staining reflected inflammatory cytokine expression, with darker staining indicating higher levels. As shown in Figure 6a, the control group showed strong and extensive positive staining, representing severe inflammation. E/Gel and MGel groups exhibited moderate staining, while the E/MGel group displayed the weakest and sparsest brown signals, indicating significantly reduced expression of pro-inflammatory cytokines.

In addition, in vivo biosafety was assessed. Histopathological analysis of major organs (Figure 6b) showed normal tissue structure and no obvious lesions such as inflammation, necrosis or fibrosis, confirming good biocompatibility of E/MGel. Serological tests (Figure 6c) further demonstrated that ALT, AST, CR and UREA levels were comparable to the normal group, with no significant hepatotoxicity or nephrotoxicity observed.

Overall, the E/MGel hydrogel effectively inhibits MRSA infection, mitigates excessive inflammation, promotes collagen deposition and angiogenesis, and significantly accelerates infected diabetic wound healing with excellent in vivo biosafety.

## 3. Conclusions

This study developed a multifunctional methacrylated hyaluronic acid (mHA)-based composite hydrogel (E/MGel) to target oxidative stress, persistent inflammation, and insufficient angiogenesis in refractory diabetic infected wounds. The hydrogel was fabricated by crosslinking mussel adhesive protein (MAP) with the mHA network and further loading epidermal growth factor (EGF), integrating the broad-spectrum antibacterial, antioxidant properties of MAP and the pro-healing function of EGF. Rheological characterization verified the elastic architecture, porous structure, and sustained protein release behavior of E/MGel. In vitro and in vivo results confirmed that the optimized hydrogel effectively eliminated reactive oxygen species, inhibited *E. coli* and MRSA infection, promoted endothelial angiogenesis, relieved local inflammation, and facilitated collagen deposition and rapid wound healing in infected diabetic wound models.

Nevertheless, this study has several inherent limitations. The in vitro antibacterial activities cannot fully reflect the therapeutic effects against mature bacterial infections in complex pathological microenvironments. Moreover, the diabetic mouse model fails to fully mimic the severe peripheral circulatory dysfunction of human diabetic patients, which may limit the evaluation of its clinical translational potential. Additionally, the in vivo degradation, metabolic characteristics and long-term biosafety of E/MGel still require systematic investigation. Future studies will adopt high-fidelity animal models with impaired peripheral circulation to further validate its antibacterial and wound-repair efficacy, and comprehensively explore its in vivo metabolic behavior and biosafety. Overall, this MAP-based hydrogel platform provides a feasible and versatile strategy for the design and fabrication of advanced dressings for refractory diabetic infected wounds.

## 4. Materials and Methods

### 4.1. Materials

Hyaluronic acid (HA, >95%, molecular weight 90,000) was obtained from Shanghai Mcline Biochemical Co., Ltd. (Shanghai, China). Mussel adhesive protein (MAP, molecular weight 100 kDa) was purchased from Jiangsu Jinpunoan Biotechnology Co., Ltd. (Nanjing, China). 4-(2-hydroxyethoxy)phenyl-(2-hydroxy-2-propyl)ketone (Irgacure 2959) was purchased from Merck (Darmstadt, Germany). Epidermal Growth Factor (EGF, recombinant synthetic, molecular weight 6.2 kDa) was purchased from Suzhou Nearshore Protein Technology Co., Ltd. (Suzhou, China). The Human EGF ELISA Kit (Catalog No. ELK10240) was purchased from ELK Biotechnology (Wuhan, China). H_2_O_2_ (30%) was purchased from Shanghai Hushi (Shanghai, China). The hydroxyl radical detection kit and superoxide anion detection kit were both purchased from Solarbio (Beijing, China). The reactive oxygen species (ROS) detection kit (DCFH-DA) was purchased from Shanghai Beyotime Biological Reagents Co., Ltd. (Shanghai, China). *E. coli* and MRSA were purchased from the Culture Collection Center of the Institute of Microbiology, Chinese Academy of Sciences (Beijing China). L929 and HUVEC cells were obtained from National Collection of Authenticated Cell Cultures, Chinese Academy of Sciences (Shanghai, China). DMEM basal medium, 1640 basal medium, monoclonal antibodies, and fetal bovine serum were provided by Jiangsu Senbeiga Company (Nanjing, China).

### 4.2. Synthesis of mHA

Methacrylated hyaluronic acid (mHA) was synthesized via esterification under alkaline conditions according to a previously reported method with minor modifications [45]. Briefly, 200 mg of HA was fully dissolved in 10 mL deionized water to form a homogeneous solution. Then, 85 μL methacrylic anhydride was added dropwise under ice-bath conditions, and the pH of the mixture was maintained at 8–9 by titration with 1 mol/L NaOH solution. The reaction was carried out with continuous stirring for 24 h at room temperature. The product was precipitated in pre-cooled anhydrous ethanol at a volume ratio of 10:1 (ethanol: aqueous solution), washed three times with anhydrous ethanol to remove unreacted impurities, dissolved in deionized water, and lyophilized to obtain mHA. The chemical structure of mHA was verified by ^1^H nuclear magnetic resonance (^1^H NMR) spectroscopy, and the double-bond grafting ratio was calculated by peak integration.

### 4.3. Preparation of Hydrogels

A precursor solution was prepared by dissolving mHA (10 mg/mL), MAP (10 mg/mL) and EGF (12.5 μg/mL) in 0.1% (*w*/*v*) Irgacure 2959 aqueous solution. These were mixed uniformly and cross-linked under UV light (365 nm, 1–5 min, 10–50 Mw/cm^2^) to form four types of hydrogels: mHA hydrogel (Gel), EGF-loaded hydrogel (E/Gel), MAP-loaded hydrogel (MGel), and EGF/MAP co-loaded composite hydrogel (E/MGel).

### 4.4. In Vitro EGF Release Study

EGF release from E/MGel and E/Gel hydrogels was evaluated. Each hydrogel (200 μL, *n* = 3) was incubated in 2 mL of release medium (PBS, pH 7.4, 0.1% BSA, 0.02% NaN_3_) at 37 °C, 100 rpm. At predetermined time points (0, 0.5, 1, 2, 4, 8, 12, 24, 48, and 72 h), the entire medium was collected and replaced with fresh medium.

EGF concentration was measured using an ELISA kit according to the manufacturer’s instructions. Cumulative release was calculated as follows:

$$\mathrm{Cumulative}\;\mathrm{release}\;\left(\%\right)=\frac{\sum (C_{i}\times 2\;\mathrm{mL})}{2.5\mu g}\times 100\%$$

where $C_{i}$ (ng/mL) is the EGF concentration at time point i, and 2.5 μg is the initial EGF loading per hydrogel.

### 4.5. Rheological Properties

Rheological characterization of E/MGel was performed using a rotational rheometer. After blue-light crosslinking, the hydrogel was transferred to the platform. Dynamic frequency sweep tests were conducted at 37 °C with a strain of 1% and a frequency range of 0.1–10 Hz. The storage modulus (G′) and loss modulus (G″) were recorded to evaluate the viscoelastic behavior.

### 4.6. Scanning Electron Microscopy (SEM)

The microstructure of lyophilized E/MGel was observed by SEM. The hydrogel was freeze-dried, brittle-fractured in liquid nitrogen, and sputter-coated with platinum. The internal porous morphology, pore size, and three-dimensional network structure were observed and recorded under an accelerating voltage.

### 4.7. Swelling Behavior

The swelling ratio of E/MGel was measured by immersing the hydrogel in PBS at room temperature. At predetermined time intervals (0.5, 1, 2, 4, 8, 12, 24, and 72 h), the hydrogel was removed, wiped gently to remove surface water, and weighed. The swelling ratio was calculated based on the weight change before and after immersion.

### 4.8. In Vitro Degradation Assay

The degradation behavior of hydrogels was evaluated using a dry weight method. Two types of hydrogels, Gel and E/MGel, were prepared. All samples were frozen at −80 °C and lyophilized to obtain the initial dry weight (W_0_). Subsequently, the samples were incubated in 10 mL of PBS (pH 7.4) with 100 U/mL hyaluronidase at 37 °C under constant shaking (100 rpm). At predetermined time points (12, 24, 48, and 72 h), the hydrogels were retrieved, rinsed with deionized water, lyophilized again, and weighed (W_t_). The remaining mass fraction was calculated as (W_t_/W_0_) × 100%.

### 4.9. Bacterial Culture

MRSA and *E. coli* were used as model strains. Bacteria were cultured in LB medium at 37 °C with shaking at 180 rpm until the logarithmic phase. The bacterial suspension was adjusted to OD_600_ = 0.1 for subsequent experiments. Two standard bacterial strains were used in this study: *E. coli* (ATCC 25922) and MRSA (ATCC 43300). All strains were cultured in Luria–Bertani (LB) medium at 37 °C with shaking at 180 rpm until the logarithmic phase.

### 4.10. Antibacterial Activity of MAP

The antibacterial activity of MAP at different concentrations (0.01–10 mg/mL) against MRSA and *E. coli* was evaluated using the plate counting method. Bacterial suspensions were incubated with MAP for 4 h at 37 °C, diluted, and spread on LB agar plates. After incubation, colonies were counted, and the antibacterial rate was calculated according to the following formula.

Antibacterial rate (%) = (CFUcontrol − CFUsample)/CFUcontrol × 100%

### 4.11. Antibacterial Activity of Hydrogels

The antibacterial performance of Gel, E/Gel, MGel, and E/MGel was evaluated using the inhibition zone method. Bacterial suspension was evenly spread on LB agar plates. Wells were punched in the plates, and hydrogel samples were placed into the wells. After incubation at 37 °C, the diameter of the inhibition zone was measured and recorded.

### 4.12. Antibacterial Mechanism Assay

The antibacterial mechanism of MAP was evaluated by measuring conductivity and absorbance at 260 nm (A_260_) of nucleic acid leakage. MRSA and *E. coli* were prepared as described in Section 4.9. Bacterial suspensions were mixed with the following samples (1 mL bacteria + 200 μL hydrogel or 10 μL polymyxin B): (i) PBS (blank control); (ii) Gel (negative control); (iii) MGel; (iv) polymyxin B (0.1 mg/mL, positive control). All mixtures were incubated at 37 °C with shaking (100 rpm). At 0, 2, 4, 6, 8 and 12 h, an aliquot was centrifuged (10,000 rpm, 2 min) and the supernatant conductivity was measured (μS/cm). After 12 h, the supernatant A_260_ was measured using a microplate reader.

### 4.13. In Vitro Antioxidant Assay

The hydroxyl radical (·OH) scavenging capacity was determined using a commercial kit based on the Fenton reaction. The absorbance was measured at 550 nm, and the scavenging rate was calculated from the changes in absorbance. Meanwhile, the superoxide anion (·O_2_^−^) scavenging activity was measured using an AP-TEMED system, with the absorbance detected at 530 nm, and the corresponding scavenging rate was calculated in accordance with the kit instructions.

### 4.14. Hemocompatibility Evaluation

Hemolysis was tested in PBS (0.9% glucose). Hydrogels (200 μL) were extracted in 800 μL PBS (0.9% glucose, 37 °C, 24 h). Washed mouse RBCs were diluted to 2% (*v*/*v*) in PBS (0.9% glucose). Negative and positive controls were PBS (0.9% glucose) and 1% Triton X-100, respectively. After 6 h at 37 °C (gentle agitation every 30 min), centrifugation (1500 rpm, 5 min) was performed. Supernatant was read at 540 nm.

Hemolysis ratio (%) = (Asample − Anegative)/(Apositive − Anegative) × 100%. Experiments were in triplicate.

### 4.15. Cell Culture

Mouse L929 fibroblasts were cultured in DMEM medium, and human umbilical vein endothelial cells (HUVECs) were cultured in 1640 medium both supplemented with 10% fetal bovine serum and 1% penicillin–streptomycin at 37 °C in a 5% CO_2_ incubator. Cells were passaged routinely and used within 3–5 passages for experiments.

### 4.16. Cytocompatibility Assay

The cytocompatibility of hydrogel extracts was evaluated using the CCK-8 assay. L929 and HUVECs were seeded in 96-well plates and treated with hydrogel extracts under H_2_O_2_-induced oxidative stress. After 2 h incubation, CCK-8 solution was added, and the absorbance at 450 nm was measured to calculate cell viability.

### 4.17. Intracellular ROS Scavenging Assay

Intracellular ROS levels were detected using the DCFH-DA fluorescent probe. L929 cells were incubated with DCFH-DA, treated with hydrogels, and observed under a fluorescence microscope. The fluorescence intensity was analyzed to evaluate intracellular ROS scavenging ability.

### 4.18. Tube Formation Assay

The pro-angiogenic capacity was assessed by HUVEC tube formation assay on Matrigel. HUVECs were seeded on the Matrigel-coated plate and incubated with hydrogel extracts. After 4 h, tube formation was imaged, and the total tube length, node number, and segment number were quantitatively analyzed using ImageJ software (ImageJ 1.53c).

### 4.19. Diabetic Mouse Model

Male ICR mice (20–24 g) were housed under standard conditions. After adaptive feeding, diabetes was induced by intraperitoneal injection of STZ (40 mg/kg) for 5 consecutive days. Fasting blood glucose levels were measured every 3 days; mice with two consecutive blood glucose values ≥ 16.7 mmol/L were considered diabetic.

### 4.20. Infected Wound Model and Treatment

A full-thickness circular skin defect (10 mm in diameter) was created on the back of diabetic mice and infected with MRSA. Mice were randomly divided into four groups (*n* = 5): control (PBS treatment), E/Gel, MGel, and E/MGel. Wounds were photographed on days 0, 3, 7, and 14, and the wound closure rate was calculated. On day 3, wound bacteria were collected, diluted, and cultured to evaluate in vivo antibacterial efficacy.

### 4.21. Histological and Immunohistochemical Analysis

On day 14, mice were sacrificed, and wound tissues were harvested, fixed, dehydrated, embedded, and sectioned. H&E and Masson’s trichrome staining were performed to observe histological repair and collagen deposition. Immunohistochemical staining was used to detect IL-6 and TNF-α expression. Immunofluorescent staining for CD31 was performed to evaluate neovascularization.

### 4.22. In Vivo Safety Evaluation

Major organs (heart, liver, spleen, lung, kidney) were harvested and stained with H&E for histological observation. Serum was collected to measure liver and kidney function indicators, including ALT, AST, UREA, and CR, using an automatic biochemical analyzer.

### 4.23. Statistical Analysis

All experiments were performed at least in triplicate. Data are expressed as mean ± standard deviation (SD). Statistical analysis was performed using one-way analysis of variance (ANOVA). A value of *p* < 0.05 was considered statistically significant.

## Data Availability

The data presented in this study are openly available in article.

## Supplementary Materials

The following supporting information can be downloaded at: https://www.mdpi.com/article/10.3390/gels12060492/s1, Figure S1: ^1^H NMR spectra of HA and mHA. The characteristic peaks of methacryloyl protons (5.75 and 6.19 ppm) are indicated; Figure S2: Time-dependent changes in supernatant conductivity of (A) MRSA and (B) *E. coli* after treatment with PBS, Gel, and MGel; Figure S3: Nucleic acid leakage (absorbance at 260 nm, A_260_) from (A) MRSA and (B) *E. coli* under different treatment.

## References

[1] Zhang Z.Y., Zhao Z.D., Huang X.Y., Zhou L.F., Jiang X., Wu H.L., Liu C.S., Huang K., Wen J.L., Liu Y.C. (2025). Galectin-3-integrin α5β1 phase separation disrupted by advanced glycation end-products impairs diabetic wound healing in rodents. Nat. Commun..

[2] Gallagher K.A., Mills J.L., Armstrong D.G., Conte M.S., Kirsner R.S., Minc S.D., Plutzky J., Southerland K.W., Tomic-Canic M., American Heart Association Council on Peripheral Vascular Disease (2024). Current Status and Principles for the Treatment and Prevention of Diabetic Foot Ulcers in the Cardiovascular Patient Population: A Scientific Statement from the American Heart Association. Circulation.

[3] Yu L.J., Sun X.X., Zhang X.Y., Sun J., He Z.G., Li J., Luo C., Zhang S.W. (2026). Nanoenzyme-engineered hydrogels reprogram wound microenvironment to accelerate diabetic wound healing. Coord. Chem. Rev..

[4] Li G.J., Zhang K.K., Wei C.X., Yu R.E., Ouyang J., Yao Y.T., Lin Y., Xu H. (2026). Reactive oxygen species signaling promotes lesion development in endometriosis through activation of the CHK1/SGK1 pathway. Sci. Bull..

[5] Zhou Q.L., Zhuang Y., Deng X.L., Jiang W.D., Wang X.D., Yuan C.Y., Lin K.L. (2026). Hydrogel-Based ROS-Regulating Strategy: Reprogramming the Oxidative Stress Imbalance in Advanced Diabetic Wound Repair. Adv. Mater..

[6] Yang H., Jia X.K., Wang T., Li J.G., Geng W., Adeli M., Ma T., Gao Y., Cheng C., Zhao W.F. (2026). Lattice-Reconstructed Ru-Clusters on FeOOH-Based Self-Adaptive Artificial Peroxisome with Programmed ROS Regulation for Infectious and Inflammatory Chronic Wounds. Adv. Mater..

[7] Zheng Y., Wang M.Y., Zhang X.E., Wu Z.M., Gao L. (2025). A bacteria-responsive nanoplatform with biofilm dispersion and ROS scavenging for the healing of infected diabetic wounds. Acta Biomater..

[8] Wang H., He P.Y., Tang G.L., Qi F.Y., Lu X., Zhang M.X., Zheng R.Z., Li X.H., Shi Z.J., Zhang Y.P. (2026). Skin repairing procedure inspired polypyrrole/bacterial cellulose/platelet rich plasma composite hydrogel as diabetes wound dressing. J. Bioresour. Bioprod..

[9] Wang S., Liang J.H., Ding R., Zhao W.H., Zhang J.H., Peng P.D., Chai J., Yan Y.B., Li P. (2025). Hierarchical ROS-scavenging platform breaks vicious cycle of stem cell senescence, angiogenesis arrest, and immune dysregulation in diabetic wounds. J. Control. Release.

[10] Yu Z.X., Li M.H., Yang L., Liu H., Ding G.Y., Ma S.N., Liu L., Dong S.J. (2024). Enhancing diabetic wound healing: A two-pronged approach with ROS scavenging and ROS-independent antibacterial properties. Nano Today.

[11] Ding J., Jiang J., Tian Y., Su B.R., Zeng M.Z., Wu C.H., Wei D., Sun J., Luo H.R., Fan H.S. (2024). Temperature-Responsive Hydrogel System Integrating Wound Temperature Monitoring and On-demand Drug Release for Sequentially Inflammatory Process Regulation of Wound Healing. ACS Appl. Mater. Interfaces.

[12] Zhang Y.Y., Liu L., Guan C.Y., Xu C.L., Liu Y., Abdullah M., Qi Y., Nie J.H., Meng K., Li M.Z. (2026). Aloe extract-loaded cationized silk fibroin/oxidized hyaluronic acid injectable self-healing hydrogel for diabetic wound healing. Int. J. Biol. Macromol..

[13] Dong L.Z., Zhang W., Ren M., Li Y.X., Wang Y.L., Zhou Y.R., Wu Y.L., Zhang Z.J., Di J.T. (2023). Moisture-Adaptive Contractile Biopolymer-Derived Fibers for Wound Healing Promotion. Small.

[14] Dai L.L., Geng Y., Ding X.F., Zhang Z.K., Lai C.H., Zhang D.H., Xia C.L., Lai Y.X. (2025). Highly stretchable, self-adhesive, and biocompatible cellulose/chitosan based double network hydrogel for wound dressing. Carbohydr. Polym..

[15] Sun L.F., Zhou J.Y., Lai J.Y., Zheng X., Wang H.Z., Lu B., Huang R.S., Zhang L.M. (2024). Novel Natural Polymer-Based Hydrogel Patches with Janus Asymmetric-Adhesion for Emergency Hemostasis and Wound Healing. Adv. Funct. Mater..

[16] Zhang H.D., Liang Q., Ji Y.X., Chen Q., Jiang W.A., Zhang D.H., Wu Y.M., Yu L.M., Chen W., Liu R.H. (2025). Facile fabrication of antioxidative and antibacterial hydrogel films to accelerate infected diabetic wound healing. Bioact. Mater..

[17] Liu Y.H., Yang X.X., Wu K.F., Feng J.Y., Zhang X., Li A., Cheng C., Zhu Y.Z., Guo H., Wang X.L. (2025). Skin-Inspired and Self-Regulated Hydrophobic Hydrogel for Diabetic Wound Therapy. Adv. Mater..

[18] Schaly S., Islam P., Prakash S. (2024). Alginate-chitosan hydrogel formulations for VEGFA expressed baculovirus delivery promoting angiogenesis for wound healing and revascularization. Eur. Heart J..

[19] Bolaños-Cardet J., Pepio-Tárrega B., Saiz-Poseu J., Lopez-Moral A., Ullah F., Yuste V.J., Ruiz-Molina D., Suárez-García S. (2026). The Redox Properties of Polyphenols and Their Role in ROS Generation for Biomedical Applications. Angew. Chem. Int. Ed..

[20] Guo Z.C., Jiang H., Song A.G., Liu X.H., Wang X.M. (2024). Progress and challenges in bacterial infection theranostics based on functional metal nanoparticles. Adv. Colloid Interface Sci..

[21] Abbasi A., McCall A., Jiang Z.W., Leblanc B.W., Shukla A. (2026). Bacterial enzyme-responsive hydrogels for triggered delivery of antibiotics to infected wounds. Sci. Adv..

[22] Pan Y.J., Xia M.Y., Luo J., Lu S. (2025). Resveratrol Promotes Wound Healing by Enhancing Angiogenesis via Inhibition of Ferroptosis. Food Sci. Nutr..

[23] Wu J.L., Deng L., Yin L., Mao Z.R., Gao X.Q. (2023). Curcumin promotes skin wound healing by activating Nrf2 signaling pathways and inducing apoptosis in mice. Turk. J. Med. Sci..

[24] Pan Y.Y., Lou J.Y., Wang Y.Q., Dai R.F., Zou M.W., Ma D.N., Chen S.Z., Zhou J.H., Chen B.Y., Jiang X.Q. (2026). A ROS scavenging multifunctional hydrogel crosslinked with EGCG promotes the healing of pressure injuries via Nrf2 pathway. Chem. Eng. J..

[25] Mottaghitalab F., Yazdi M.K., Saeb M.R., Baczek T., Farokhi M. (2024). Green and sustainable hydrogels based on quaternized chitosan to enhance wound healing. Chem. Eng. J..

[26] Liu X.Q., Sun Y.M., Wang J., Kang Y.Y., Wang Z.L., Cao W.B., Ye J., Gao C.Y. (2024). A tough, antibacterial and antioxidant hydrogel dressing accelerates wound healing and suppresses hypertrophic scar formation in infected wounds. Bioact. Mater..

[27] Yang J.H., Wang Z.Y., Liang X.B., Wang W.Y., Wang S.G. (2024). Multifunctional polypeptide-based hydrogel bio-adhesives with pro-healing activities and their working principles. Adv. Colloid Interface Sci..

[28] Li C.C., Du L.W., Xiao Y.Y., Fan L., Li Q.L., Cao C.Y. (2025). Multi-active phlorotannins boost antimicrobial peptide LL-37 to promote periodontal tissue regeneration in diabetic periodontitis. Mater. Today Bio.

[29] Wu S.H., Xia X., Zhou R.H., Zhao H. (2025). Hydrogel-enabled ROS-GSH modulation for sustained copper-mediated chemodynamic therapy of oral squamous cell carcinoma. J. Control. Release.

[30] Dan A., Singh H., Darban Z., Yadav I., Munawar J., Shah S.A., Lone M.A., Shahabuddin S., Hassan S., Bashir S.M. (2025). Therapeutic β-carotene-loaded inulin-based multifunctional hydrogel for full-thickness skin wounds with polymicrobial infections. Carbohydr. Polym..

[31] Ye Y., Zhong W.Z., Luo R.F., Wen H.Z., Ma Z.Y., Qi S.S., Han X.Q., Nie W.B., Chang D.G., Xu R.C. (2024). Thermosensitive hydrogel with emodin-loaded triple-targeted nanoparticles for a rectal drug delivery system in the treatment of chronic non-bacterial prostatitis. J. Nanobiotechnol..

[32] Wang W.Y., Liu C.L., Sun Y. (2025). Multifunctional mPDA@Mel-AB/BG hydrogels: Integrating ROS scavenging, inflammation suppression, and cartilage repair for osteoarthritis therapy. J. Control. Release.

[33] Chen J.S., Han L.B., Liu J.F., Zeng H.B. (2023). Mussel-Inspired Adhesive Hydrogels: Chemistry and Biomedical Applications. Chin. J. Chem..

[34] Tiu B.D.B., Delparastan P., Ney M.R., Gerst M., Messersmith P.B. (2020). Cooperativity of Catechols and Amines in High-Performance Dry/Wet Adhesives. Angew. Chem. Int. Ed..

[35] Chen J.S., Zeng H.B. (2022). Mussel-Inspired Reversible Molecular Adhesion for Fabricating Self-Healing Materials. Langmuir.

[36] Deepankumar K., Guo Q., Mohanram H., Lim J., Mu Y.G., Pervushin K., Yu J., Miserez A. (2022). Liquid-Liquid Phase Separation of the Green Mussel Adhesive Protein Pvfp-5 is Regulated by the Post-Translated Dopa Amino Acid. Adv. Mater..

[37] Das S., Rodriguez N.R.M., Wei W., Waite J.H., Israelachvili J.N. (2015). Peptide Length and Dopa Determine Iron-Mediated Cohesion of Mussel Foot Proteins. Adv. Funct. Mater..

[38] Li Y.R., Cheng J., Delparastan P., Wang H.Q., Sigg S.J., DeFrates K.G., Cao Y., Messersmith P.B. (2020). Molecular design principles of Lysine-DOPA wet adhesion. Nat. Commun..

[39] Cheong H., Kim J., Kim B.J., Kim E., Park H.Y., Choi B.H., Joo K.I., Cho M.L., Rhie J.W., Lee J.I. (2019). Multi-dimensional bioinspired tactics using an engineered mussel protein glue-based nanofiber conduit for accelerated functional nerve regeneration. Acta Biomater..

[40] Yamakawa S., Hayashida K. (2019). Advances in surgical applications of growth factors for wound healing. Burn. Trauma.

[41] Wang S.Q., Liu Y.H., Wang X.S., Chen L.Q., Huang W., Xiong T.N., Wang N.Y., Guo J.P., Gao Z.G., Jin M.J. (2024). Modulating macrophage phenotype for accelerated wound healing with chlorogenic acid-loaded nanocomposite hydrogel. J. Control. Release.

[42] Yang Y., Luo R.Z., Chao S.Y., Xue J.T., Jiang D.J., Feng Y.H., Guo X.D., Luo D., Zhang J.P., Li Z. (2022). Improved pharmacodynamics of epidermal growth factor via microneedles-based self-powered transcutaneous electrical stimulation. Nat. Commun..

[43] Hao M., Zhang Z.X., Liu C., Tian Y., Duan J.Z., He J.L., Sun Z.Y., Xia H., Zhang S., Wang S.H. (2021). Hydroxyapatite Nanorods Function as Safe and Effective Growth Factors Regulating Neural Differentiation and Neuron Development. Adv. Mater..

[44] Itzhakov R., Eretz-Kdosha N., Silberstein E., Alfer T., Gvirtz R., Fallik E., Ogen-Shtern N., Cohen G., Poverenov E. (2023). Oligochitosan and oxidized nucleoside-based bioderived hydrogels for wound healing. Carbohydr. Polym..

[45] Schuurmans C.C.L., Mihajlovic M., Hiemstra C., Ito K., Hennink W.E., Vermonden T. (2021). Hyaluronic acid and chondroitin sulfate (meth)acrylate-based hydrogels for tissue engineering: Synthesis, characteristics and pre-clinical evaluation. Biomaterials.

