# Supplementary material for: Mussel Adhesive Protein/Hyaluronic Acid Hydrogels for EGF Delivery and MRSA-Infected Diabetic Wound Repair
Source: Gels. 2026 Jun 2;12(6):492. doi: 10.3390/gels12060492 (PMC13298544; doi:10.3390/gels12060492)
Supplement: Supplementary file 1 [file gels-12-00492-s001.zip › gels-4321090-supplementary.pdf]

Supplementary Materials

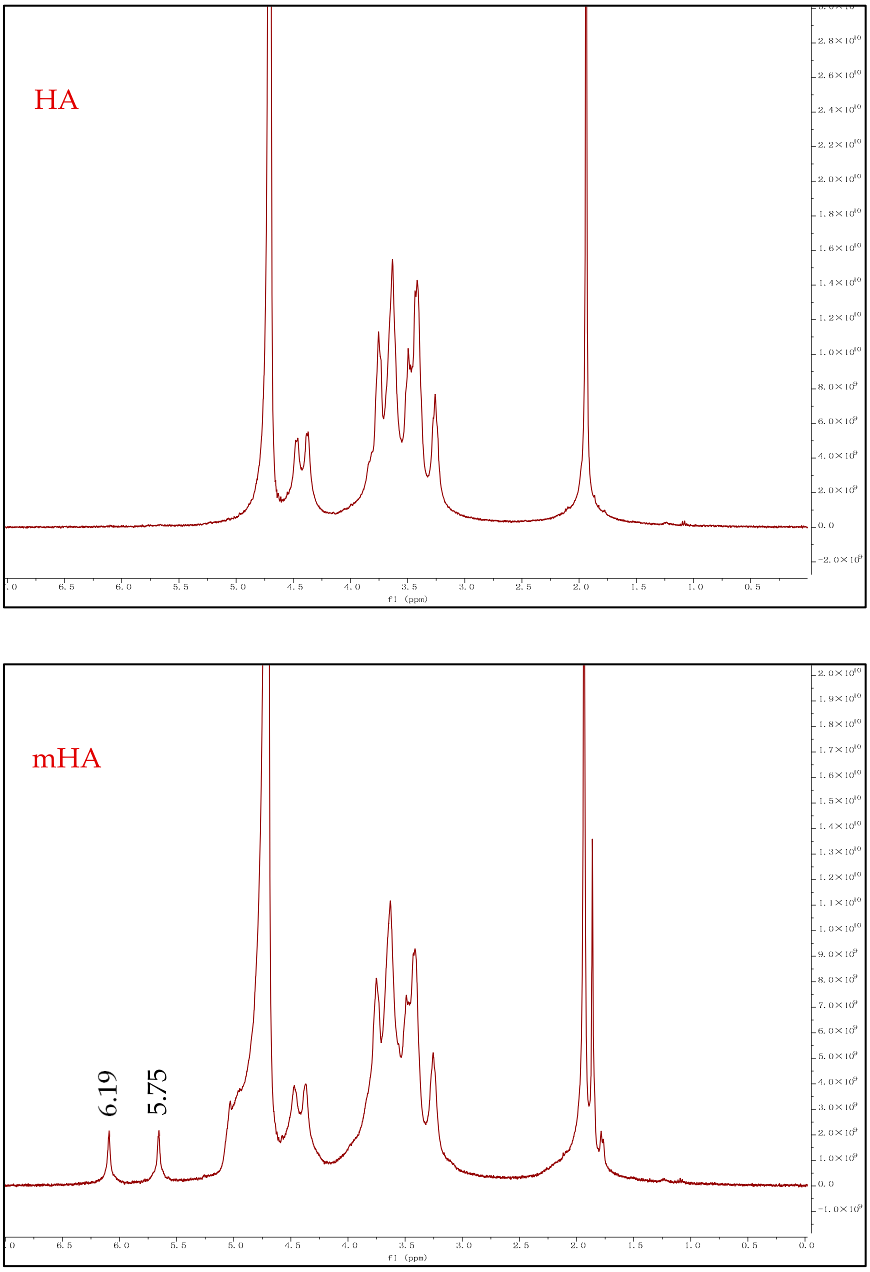

Figure S1. ^{1}H NMR Spectra of HA and mHA.

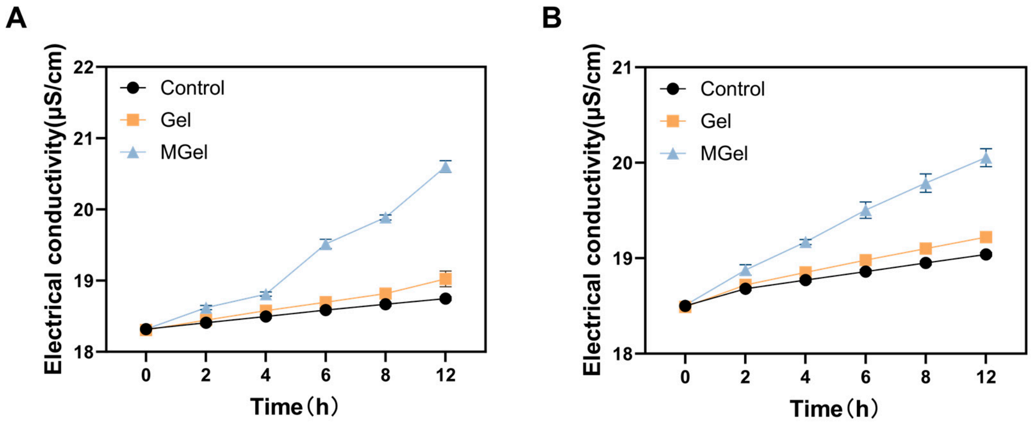

Figure S2. Time-dependent changes in supernatant conductivity of (A) MRSA and (B) *E. coli.* Data are presented as mean ± SD ($n = 3$).

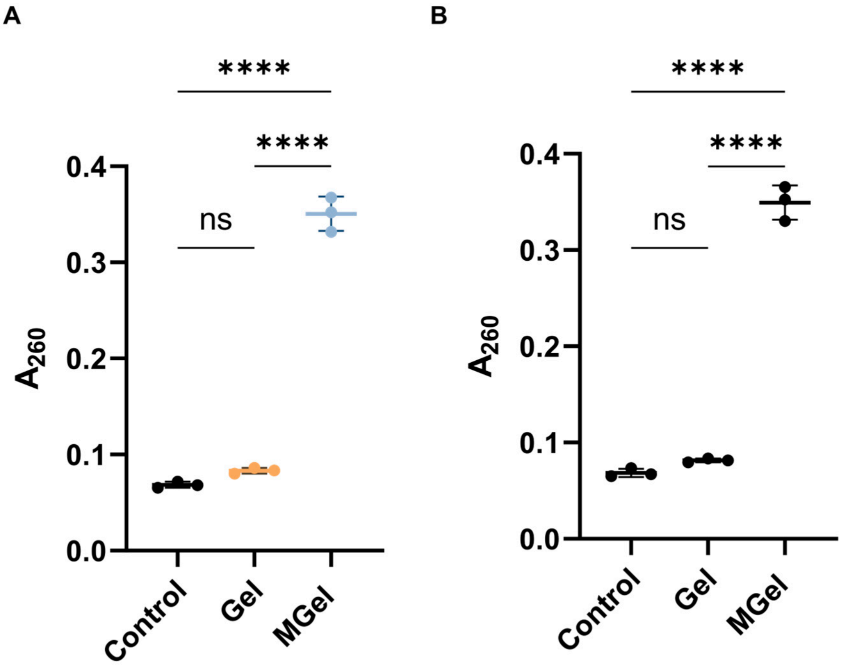

Figure S3. Nucleic acid leakage (absorbance at 260 nm, A_{260}) from (A) MRSA and (B) *E. coli.* Data are presented as mean ± SD ($n = 3$).
